# Genomic Analyses Reveal Broad Impact of miR-137 on Genes Associated with Malignant Transformation and Neuronal Differentiation in Glioblastoma Cells

**DOI:** 10.1371/journal.pone.0085591

**Published:** 2014-01-22

**Authors:** Saleh Tamim, Dat T. Vo, Philip J. Uren, Mei Qiao, Eckart Bindewald, Wojciech K. Kasprzak, Bruce A. Shapiro, Helder I. Nakaya, Suzanne C. Burns, Patricia R. Araujo, Ichiro Nakano, Agnes J. Radek, Scott Kuersten, Andrew D. Smith, Luiz O. F. Penalva

**Affiliations:** 1 Children's Cancer Research Institute, University of Texas Health Science Center at San Antonio, San Antonio, Texas, United States of America; 2 Molecular and Computational Biology Section, Division of Biological Sciences, University of Southern California, Los Angeles, California, United States of America; 3 Basic Science Program, SAIC-Frederick, Inc., Center for Cancer Research Nanobiology Program, Frederick National Laboratory for Cancer Research, Frederick, Maryland, United States of America; 4 Center for Cancer Research Nanobiology Program, National Cancer Institute, Frederick, Maryland, California; 5 Department of Clinical Analyses and Toxicology, Institute of Pharmaceutical Sciences, University of São Paulo, São Paulo, Brazil; 6 Department of Neurological Surgery, James Comprehensive Cancer Center, The Ohio State University, Columbus, Ohio, United States of America; 7 Epicentre (An Illumina Company), Madison, Wisconsin, United States of America; 8 Department of Cellular and Structural Biology, University of Texas Health Science Center at San Antonio, San Antonio, Texas, United States of America; Sun Yat-sen University Medical School, China

## Abstract

miR-137 plays critical roles in the nervous system and tumor development; an increase in its expression is required for neuronal differentiation while its reduction is implicated in gliomagenesis. To evaluate the potential of miR-137 in glioblastoma therapy, we conducted genome-wide target mapping in glioblastoma cells by measuring the level of association between PABP and mRNAs in cells transfected with miR-137 mimics vs. controls via RIPSeq. Impact on mRNA levels was also measured by RNASeq. By combining the results of both experimental approaches, 1468 genes were found to be negatively impacted by miR-137 – among them, 595 (40%) contain miR-137 predicted sites. The most relevant targets include oncogenic proteins and key players in neurogenesis like c-KIT, YBX1, AKT2, CDC42, CDK6 and TGFβ2. Interestingly, we observed that several identified miR-137 targets are also predicted to be regulated by miR-124, miR-128 and miR-7, which are equally implicated in neuronal differentiation and gliomagenesis. We suggest that the concomitant increase of these four miRNAs in neuronal stem cells or their repression in tumor cells could produce a robust regulatory effect with major consequences to neuronal differentiation and tumorigenesis.

## Introduction

miRNAs (microRNAs) have been shown to be critical players in the nervous system and are implicated in multiple processes including neurogenesis, as well as neurological disorders, neurodegenerative diseases and brain tumors. In the context of glioblastomas, miRNA signatures were used to re-classify tumors and assess prognosis [1] and to build networks to define novel oncogenic pathways [2], [3]. A small group of miRNAs that includes miR-7, miR-124, miR-128 and miR-137 has been shown by numerous studies to be present at the interesting intersection between neurogenesis and brain tumor development. More specifically, an increase in their expression seems to be required for neuronal differentiation while their down regulation is frequently observed in gliomas and associated with disease progression [4]–[6]. We focused our study on miR-137 since it is the most well-characterized amongst the four above cited miRNAs in neurogenesis and tumorigenesis, being also an important tumor suppressor miRNA in other malignancies.

The expression of miR-137 was observed to be dramatically up-regulated during differentiation of A94 neuronal stem cells (NSC) [7] and mouse embryonic stem cells (mESCs) [8] and its levels of expression were determined to be significantly higher in isolated primary neurons compared with aNSCs [7] and in differentiated vs. undifferentiated neuroblastoma cells [9]. miR-137 affects neuronal dendritic development [7] and *in utero* electroporation of miR-137 in mouse embryonic brains produced premature differentiation [10]. Similarly, transfection of miR-137 in mouse neural stem cells (mNSCs) produced a five-fold increase in the number of differentiated cells once growth factors were removed from the media [6].

With regard to tumorigenesis, differential expression of miR-137 was not only observed in comparisons between normal brain and tumor tissue but also in low vs. high grade glioma, suggesting that low miR-137 could be related to poor prognosis [6], [11]. In addition, miR-137 expression was found to be significantly down-regulated in a cohort of 35 oligodendroglial tumors compared with normal brains. Lower miR-137 expression was associated with both shorter progression-free and overall survival [12]. Transfection of miR-137 mimics in glioma cells decreased proliferation, invasion and anchorage-independent-growth, produced cell cycle arrest in G_0_/G_1_ phase and affected their growth as xenografts [6], [11], [12]. Other studies have found that miR-137 inhibits the stemness of glioma stem cells by targeting RTVP-1 [13].

The participation of miR-137 in tumorigenesis is not restricted to glioblastoma; miR-137 has been extensively studied in colon cancer where its expression is inhibited via promoter hypermethylation. Similar to what has been observed in glioblastoma cells, restoration of miR-137 reduced cell proliferation of colon cancer lines HCT116 and RKO [14]. Regulation of miR-137 expression via promoter hypermethylation is perhaps a common mechanism as it was also established in oral cancer, gastric cancer and squamous cell carcinoma of head and neck [15]–[18]. Uveal melanoma is another tumor type affected by miR-137 where its expression is lower in uveal melanoma cell lines when compared to uveal melanocytes. Ectopic expression of miR-137 in melanoma cells induced G_1_ cell cycle arrest and a decrease in cell growth [19]. A connection between miR-137 and breast cancer has been suggested based on its regulation of orphan nuclear receptor ERRα, a prognostic factor of poor clinical outcome. Down-regulation of ERRα mediated by miR-137 impaired proliferative and migratory capacity of breast cancer cells [20]. In addition, ectopic expression of miR-137 in lung cancer cells induced G_1_ cell cycle arrest and decreased cell growth *in vivo* and *in vitro* [21]. Clearly, miR-137 is an important player in a diverse set of cancer systems and further understanding of its mechanism of action and its mRNA targets are warranted.

Several miR-137 targets have been identified in the context of the neuronal system including lysine-specific demethylase (LSD1) [10], RTVP-1 [13], KDM1A [22], Mind Bomb-1 (Mib1) [7], COX-2 [11], the histone methyltransferase Ezh2 [23], the cell cycle regulator CDK6 [6], the oncogenic RNA binding protein Musashi1 (Msi1) [9], CSE1 chromosome segregation 1-like (CSE1L) [12] and Jarid1b, a histone H3 Lys4 demethylase [8]. However, determining the genome-wide impact miR-137 transfection would have on glioblastoma cells is a mandatory step to establish its potential as a therapeutic agent. We have conducted genomic analyses in glioblastoma cells including the usage of a novel approach inspired by the recently described mechanism of miRNA action via PABP (poly A binding protein) and the poly A tail [24], [25]. Our results led to the identification of 595 targets of miR-137, comprising important oncogenic proteins such as c-KIT, AKT2, YBX1, CD24, CDC42 and TGFβ2. We also determined that miR-137 potentially shares a large portion of its targets with miR-7, miR-124 and miR-128, indicating that their absence as a group in neuronal cells could constitute an important contribution to gliomagenesis.

## Materials and Methods

### Cell growth and transfection

U251 and U343 glioblastoma cells (ATCC) were grown in DMEM medium with 10% FBS. Mir-137 mimic or NC mimic (Qiagen) were transfected into cells in 10 cm dishes using Lipofectamine RNAiMax reagent (Invitrogen) by reverse transfection according to the manufacturer's instructions; transfection complexes were prepared and added directly to the cell at a final concentration of 25 nM. Cells were harvested after 48 hours. Transfection efficiency was determined by qRT-PCR. The cDNA synthesis and qRT-PCR were done by using Taqman small RNA assays kit (Life Technologies).

### Cell proliferation assay

U251 cells (750cell/well) were transfected with miRNA mimics at 25 nM and grown in a 96-well Essen ImageLock cell culture plate (Essen BioScience). Percentage confluence was monitored using the high definition automated imaging system from IncuCyte (Essen BioScience) following the manufacturer's direction. The experiments were performed in quadruplicate. The data was analyzed using analysis of variance and displayed as mean ± standard deviation.

### Caspase-3/-7 apoptosis assay

U251 cells were reverse transfected with miRNA mimics at 25 nM and grown in 96 well plates (1500cells/well). After incubation for 90 and 120 hours, apoptosis was assessed using Caspase-Glo 3/7 assay kit from Promega according to the manufactuer's protocol. Luminescence was measured with the Molecular Devices SpectraMax M5 microplate reader (Molecular Devices). The data was analyzed using the Student's *t*-test and displayed as mean ± standard deviation.

### Cell migration assay

To study cell migration *in vitro*, we used the *in vitro* scratch assay. U251 cells were grown in a 96-well Essen ImageLock cell culture plate (Essen BioScience) in a standard CO_2_ incubator. The cells were transfected at a low density with miRNA mimics for a period of 48 hours, to approximately 100% confluency. The floor of the wells was left uncoated. The 96-pin WoundMaker (Essen BioScience) was used to create precise and reproducible wounds in the 96-well plate. Assay plates were then equilibrated for 15 minutes within the Essen IncuCyte automated microscope system (Essen BioScience) before the first scan. The software was set to scan every 15 minutes for 4 days. The data was analyzed by using the Relative Wound Density integrated metric. At the end of the experiment, the data was inspected using analysis of variance and displayed as mean ± standard deviation.

### RNASeq and RIPSeq experiments

RIP experiments were performed according to established protocols [26], [27]. Briefly, cell extracts were prepared from U251 and U343 transfected cells (miR-137 and control mimics) in polysomal lysis buffer, diluted in NT2 buffer and incubated for 3 hours with protein A-sepharose 4Fast flow from GE (Cat.17-5280-01) conjugated with a mix of PABP antibodies (anti-PABP I and anti-iPABP produced in Jack Keene's lab). Beads were washed in NT2 buffer five times and subsequently digested with Proteinase K. RNA was extracted with phenol-chloroform, precipitated with isopropanol and kept at −80°C until usage.

Total RNA was purified from control and miR-137 transfected cells with Trizol (Life Technologies). Total RNA and RIP samples were depleted of 28S, 18S, 5.8S and 5S rRNA using RiboZero rRNA removal kit from Epicentre as per manufacters instructions. The rRNA depleted samples from U251 cells (16 µl) were then mixed with 1 µL random hexamers (500 ng) and 1 µL 10X MMLV Buffer (Epicentre), heated to 65°C for 2 minutes and placed on ice. To this mixture was added 2.5 mM DTT, 0.5 mM dNTPs, 40 units of Riboguard (Epicentre) and 50 units of EpiScript RT (Epicentre). First strand cDNA was then synthesized by incubation for 10 minutes at 25°C, 30 minutes at 42°C and 15 minutes at 70°C, then held at 4°C in a thermocycler with a heated lid. Second strand cDNA was synthesized by addition of 30 mM Tris pH 7.5, 5 mM MgCl_2_, 5 mM DTT, 0.3 mM each dNTP, 2.5 units of E. coli RNase H (Epicentre) and 20 units of E. coli DNA polymerase I (Epicentre). The mixtures were then incubated for one hour at 16°C, followed by 15 minutes at 80°C. The reactions were then purified using Zymo DNA Clean kit and the samples were eluted with 16 µL of nuclease-free water. The double-stranded cDNA was then fragmented and tagged using the Nextera DNA library kit from Epicentre as per the protocol supplied by the manufacturer and PCR amplified for 9 cycles. The samples were purified using Zymo DNA Clean kit and quantitated by using a bioanalyzer and Qubit methods, then sequenced on an Illumina GAIIx instrument for 36 cycles. After Ribo-Zero treating the RNA samples from the U343 cells, 50 ng were converted into double-stranded cDNA using the TotalScript cDNA kit (Epicentre, cat. #TSCD12924) as per the supplied protocol and purified using the Zymo DNA Clean and Concentrator kit. The cDNA was then tagmented and amplified using the Nextera DNA sample prep kit (Illumina, #FC-121-1031) and purified using the Zymo DNA clean kit. The samples were quantitated by bioanalyzer and Qubit methods, then sequenced on an Illumina GAIIx instrument for 36 cycles.

### Analysis of RNASeq and RIPSeq data

Sequencing data was aligned to hg18 using RMAP [28] and counted to a transcriptomic reference created from RefSeq by collapsing isoforms and producing one super-transcript per gene. To identify transcripts that are significantly down-regulated upon transfection of the miR-137 mimic, we constructed a two-by-two contingency table counting the number of reads (1) mapped to the transcript in the control, (2) mapped to other transcripts in the control, (3) mapped to the transcript in the miR-137 transfection and (4) mapped to other transcripts in the miR-137 transfection. We then applied Fisher's exact test to each of these tables (21,698 tables in total). We correct the resultant *p*-values for multiple hypothesis testing using the method of Benjamini and Hochberg [29], and retain each transcript that has an odds ratio of greater than 2× down-regulation with a corrected *p*-value smaller than 0.001. We repeat this process for each pairwise comparison of control and transfection (four in total), then retain only those transcripts which are identified in all four comparisons to arrive at a final high-confidence target set. The process is identical for both the Total-RNA and IP samples, and in both the U251 and U343 cells.

To ensure the highest specificity for the data analysis, we required that each target is supported in all replicates. In our analysis, we have an additional replicate for the U251 experiments, making the U251 dataset more stringent. Additionally, the sequencing depth for the U343 dataset was greater by approximately 20%, and the mapping rates were higher. These attributes ensured a higher statistical power to confidently identify more mRNA targets.

Sequence data has been deposited to Gene Expression Omnibus (GEO) [Accession Number: GSE53220].

### Analysis of miR-137 target genes

We obtained lists of genes containing miR-137 predicted sites according to predictions by TargetScan (2743 targets), miRanda (3258 targets) or PicTar (850 targets); in the case of TargetScan, both conserved and non-conserved predicted targets were considered. To determine if the experimental gene sets contain an enriched number of miR-137 predictions, we performed a hypergeometric test with each prediction tool; only genes expressed in U251 or U343 cells were used as background respectively.

### Western blot

miR-137, -124, -128, -7 mimic or control miRNA mimic were transfected into both U251 and U343 using Lipofectamine RNAiMax reagent (Invitrogen) by reverse transfection according to the manufacturer's instructions. After collection of cells for analysis, they were resuspended and sonicated in Laemmli sample buffer. A 12% and 8% SDS-PAGE gel with a 4% stacking gel was run in Tris-glycine-SDS buffer. A wet transfer procedure was carried out onto nitrocellulose membrane. After transfer, the membrane was blocked in TBS with Tween-20 and 5% milk. The different membranes were probed with mouse monoclonal anti-Neurofilament-L (Cell Signaling), mouse monoclonal anti-c-Kit (cell signaling), Rabbit polyclonal anti-YB1 (Cell Signaling), mouse monoclonal anti-Cdc42 (Santa Cruz), mouse monoclonal anti-α-tubulin (Sigma) mouse monoclonal anti-CDK6 (Cell Signaling), rabbit polyclonal anti-MIB-1 (Cell Signaling), and rabbit polyclonal anti-PARP1 (Cell Signaling). HRP-conjugated goat anti-rabbit antibody (Santa Cruz biotechnology) was used as a secondary antibody for YB1, and HRP-conjugated goat anti-mouse antibody (Zymed laboratories) were used as a secondary antibody for neurofilament-L, c-Kit, CDC-42 and α-tubulin. All the proteins were detected by using Immobilon Western chemiluminescent HRP substrate (Millipore). To evaluate Akt pathway activation, U251 cell were serum starved for 16 hours, 24 hours post-transfection and probed with anti-Akt and anti-pAkt antibodies (Cell Signaling). For PARP cleavage Western blots, 25 µM of etoposide was used as a positive control for inducing apoptosis.

All Western blots were analyzed for band intensity using ImageJ software (National Institutes of Health). The data was analyzed using arbitrary band intensity generated by ImageJ, and relative band intensity was calculated by dividing the arbitrary intensity for the target protein by the arbitrary band intensity for the endogenous control. Then, all of the data was normalized to the control experiment within each Western blot to a value of 1. All experiments were performed in triplicate. Data is shown as mean ± standard deviation. * denotes P≤0.05, ** denotes P≤0.01, and *** denotes P≤0.001.

### Quantitative reverse transcription and polymerase chain reaction

Total RNA was extracted using the TRIzol reagent (Invitrogen). Briefly, TRIzol was added to the cells for lysis and dissociation of any RNA:protein complexes. Chloroform was added for phase separation. Total RNA, located in the aqueous phase, was precipitated using isopropyl alcohol. After centrifugation, the RNA pellet was washed in 75% ethanol and resuspended in nuclease-free water.

Reverse transcription of messenger RNAs was performed using the High Capacity cDNA Reverse Transcription kit (Applied Biosystems) with random priming. For mRNA analysis, quantitative PCR was performed using Gene Expression Assays and TaqMan Gene Expression Master Mix (Applied Biosystems). Real-time PCRs were performed on a 7500 Real Time PCR System (Applied Biosystems). Data was acquired using the SDS 2.0.1 software package (Applied Biosystems), and analyzed using the 2^−ΔΔCt^ method using β-actin as an endogenous control.

### Luciferase assay

HeLa Cells were co-transfected using DharmaFECT Duo (Thermo Scientific) with 50 ng of dual luciferase reporter 3′UTR clones and 10 nM either miR-137 or control mimics. To test miR-137 sites, identified seed sequences were deleted from 3′ UTR constructs. Luciferase activity was measured 48 hr after transfection using Dual-Glo luciferase assay system (Promega). The experiments were performed in triplicate. Data was analyzed using GraphPad Prism 5 (GraphPad Software, Inc.). Data is shown as mean ± standard deviation. * denotes P≤0.05, ** denotes P≤0.01, and *** denotes P≤0.001.

### Overlap between targets of miR-137 and other miRNAs

Lists of miR-7, miR-124, and miR-128 predicted targets were generated with TargetScan (conserved and non-conserved) [30], miRanda [31], and PicTar [32]. Results were compiled and compared to the miR-137 targets identified in the RNASeq and RIPSeq experiments to determine how many putative targets are shared between miR-137 and the other three miRNAs. A hypergeometric test was used to evaluate the significance of the overlap between miR-137 and other miRNA targets.

### Bioinformatics analyses

DAVID [33], [34] was used for GO enrichment analysis. Ingenuity Pathway Analysis (http://www.ingenuity.com) was used to build a biological network based on gene-gene interactions contained in its database.

### TCGA Sample analysis

We analyzed 261 TCGA samples; sample IDs were taken from Kim et al. supplementary material [1]. Each sample was assigned to a GBM type, as defined by Kim et al. (either astrocytic, neural, neuromesenchymal, oligonueral or radial glial) [1]. Gene expression z-scores for the 595 miR137 target genes relative to normal tissue were downloaded from cBioPortal, as were z-scores for miR137 in each of the 261 samples [35], [36]. We considered miR137 to be up-regulated if its z-score relative to normal tissue was greater than 0, and down-regulated if less than 0. We determined whether a significant proportion of samples showed down-regulation using the binomial test. Our purpose here was not to test whether any individual change was significant, but rather whether a significant number of changes showed a directional bias.

## Results

### miR-137 impacts cell proliferation, apoptosis and migration

**Figure 1:**
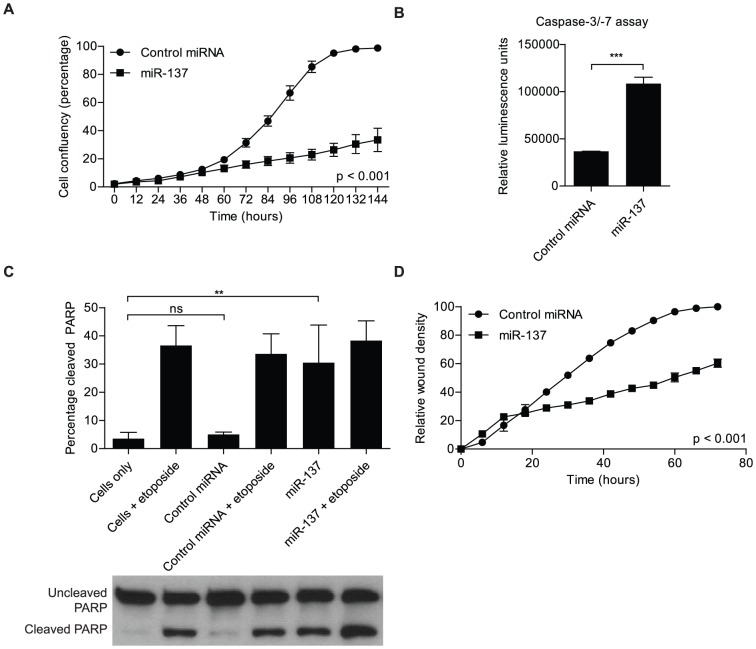
Impact of miR-137 mimics transfection on cancer relevant processes. **A**) miR-137 transfection into U251 glioblastoma cells inhibits cell proliferation (p<0.001, multiple comparison tests between groups). U251 cells were transfected with miR-137 or control miRNA and plated at a low density (500 cells per well). Cell proliferation was monitored using the Essen Bioscience IncuCyte automated microscope system and read out as percentage confluence. The experiment was monitored over a course of 6 days. The data was analyzed using analysis of variance, and the data is presented as the mean ± standard deviation. **B**) miR-137 transfection into U251 glioblastoma results in increased apoptosis, measured by caspase-3/-7 luminescent assay. Caspase-3/-7 activity, an indicator of apoptosis induction, increased after miR-137 transfection, as compared to the control miRNA transfection. Data was analyzed with Student's *t*-test and is presented as the mean ± standard deviation. *** indicates p≤0.001. **C**) miR-137 transfection into U251 glioblastoma cells results in increased apoptosis, measured by Western blot of poly(ADP) ribose polymerase (PARP) cleavage. PARP cleavage, an end event of apoptosis, increased after miR-137 transfection, as compared to the control miRNA transfection. Etoposide (25 µM) was used as an inducer of apoptosis. Data was analyzed with Student's *t*-test and is presented as the mean ± standard deviation. ** indicates p≤0.01. α-tubulin was included as an endogenous loading control. **D**) miR-137 transfection into glioblastoma cells results in decreased ability to migration (p<0.001, multiple comparison tests between groups). U251 glioblastoma cells were transfected with miR-137 or control miRNA. An *in vitro* scratch assay was used to measure the ability for cell migration. The assay was monitored using Essen Bioscience IncuCyte automated microscope system. The data was analyzed using analysis of variance, and the data is presented as the mean ± standard deviation.

miR-137 has been established as a tumor suppressor miRNA in glioblastoma and its ectopic expression has been shown to affect multiple cancer relevant processes [6], [11]. We performed cell-based assays to further examine the impact of miR-137 in glioblastoma cells. First, a non-labeled cell proliferation assay with the aid of the IncuCyte automated microscope system revealed that cell proliferation is inhibited upon transfection with miR-137, as compared to cells transfected with the control miRNA mimic (p<0.001) (**Figure 1A**). Next, we inquired whether or not miR-137 also affected apoptosis, or programmed cell death. miR-137 transfection resulted in increased caspase-3/-7 activity compared to cells transfected with the control miRNA mimic (p<0.001) (**Figure 1B**). We then followed up with a western blot to assay for poly-ADP ribose polymerase (PARP) cleavage, an end-proteolytic event that occurs in apoptosis. We utilized 25 µM of etoposide to elicit apoptosis as a positive control. We found that miR-137 transfection resulted in an increased percentage of cleaved PARP (p<0.01) (**Figure 1C**); corroborating miR-137 role in apoptosis. Another important characteristic of cancer cells is the ability to migrate, a fact that is clinically relevant in glioblastoma as many patients present secondary tumors that cross the midline. A cell migration assay with the aid of the IncuCyte established that glioblastoma cells exhibited decreased ability to migrate when cells were transfected with miR-137 as compared to cells transfected with the control miRNA mimics (p<0.001) (**Figure 1D**).

### A dual genomic approach evaluates the impact of miR-137 in glioblastoma cells

A required step to determine the potential of using miR-137 mimics as a therapeutic approach to impair glioblastoma growth is to determine its genome-wide impact. Two distinct genomic approaches were used to map the effect of miR-137 in glioblastoma cells. First, RNASeq was employed to determine changes at the mRNA level triggered by transfection of miR-137 mimics. In a second approach, we used RIPSeq to identify which mRNA species change their level of association with Poly A binding protein (PABP) upon miR-137 transfection. More specifically, mRNAs associated with PABP were immuno-precipitated and the differences in the profile of control vs. miR-137 transfected cells were assessed by next-generation sequencing – **Figure S1**. The idea behind this strategy is that PABP displays key functions in miRNA activity as recently described by Moretti et al. [24], and therefore, can act as a reporter of miRNA targeting. According to this model, mRISC ultimately disrupts the PABP-polyA interaction in target mRNAs. We expected miR-137 to disrupt the interaction between PABP and its target mRNAs, decreasing the levels of immuno-precipitated mRNAs; the read-out would be a reduction in the number of sequence reads for the target mRNAs.

**Figure 2:**
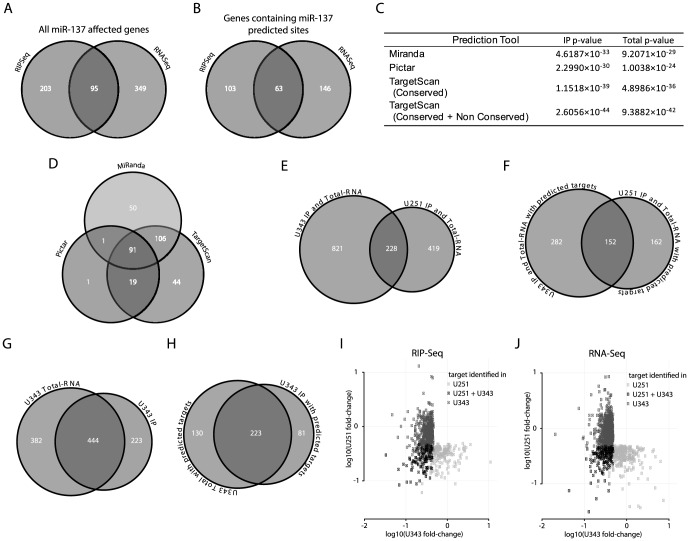
Analysis of miR-137 impact on glioblastoma expression by two genomic approaches. **A**) Venn diagram shows the number of genes affected by miR-137 mimics transfection in U251 cells obtained by RNASeq and RIPSeq approaches. **B**) Venn diagram shows the number of genes identified by our approaches containing miR-137 predicted targets according to TargetScan, Miranda or Pictar. **C**) Hypergeometric test determined the significance of results of the two approaches used to identify miR-137 targets. **D**) Venn diagram shows the distribution of genes containing miR-137 predicated sites (from B) according to prediction tools. **E**) Venn diagram showing the number of genes identified by either the RIPSeq assay or the RNASeq assay in U251 cells, U343 cells, and both cell types. **F**) As in panel E, but considering only genes that were predicted as miR137 targets by Miranda, Pictar or TargetScan. **G**) Venn diagram showing the number of genes identified as miR137 targets in U343 cells by the RIPSeq assay, the RNASeq assay and both. **H**) As in panel G, but considering only genes that were predicted as miR137 targets by Miranda, Pictar or TargetScan. **I**) The average log10-fold-change in U251 cells (y-axis) and U343 cells (x-axis) is shown for all genes identified as miR137 targets by the RIPSeq assay in either U251 or U343 cells; genes (points) are colored by whether they were identified in only U251 cells, only U343 cells, or both. **J**) As in panel I, but for the RNASeq assay.

The two approaches combined identified in U251 cells a total of 647 mRNAs down-regulated by transfection of miR-137 mimics; 95 mRNAs were identified by both RIPSeq and RNASeq methods – **Figure 2A**. Decreased mRNA binding to PABP, which is identified using RIPSeq, indicates regulation by miR-137, as compared to the control transfection. In RNASeq analysis, miR-137 affected genes were identified by the decrease in the number of reads for transcripts upon miR-137 transfection. For those targets that were identified in only one set, we compared the differences in fold change. We observed that close to half such targets show more than an average 1× greater decrease in the approach where it was identified over that where it was not – for example, a target identified in the RNASeq method with an average fold-change of 2× down compared to an average of 1× (i.e. no change) in the RIPSeq method would represent a 1× difference. This indicates that such targets are not simply close to thresholds, but rather have markedly different fold-change scores between the two methods (see **Figure S2** for more details). We suggest that differences in regulation, position and number of miR-137 targets and cross-talk with other miRNAs and RNA binding proteins might contribute to the fact that some genes were identified by one but not the other method.

**Figure 3:**
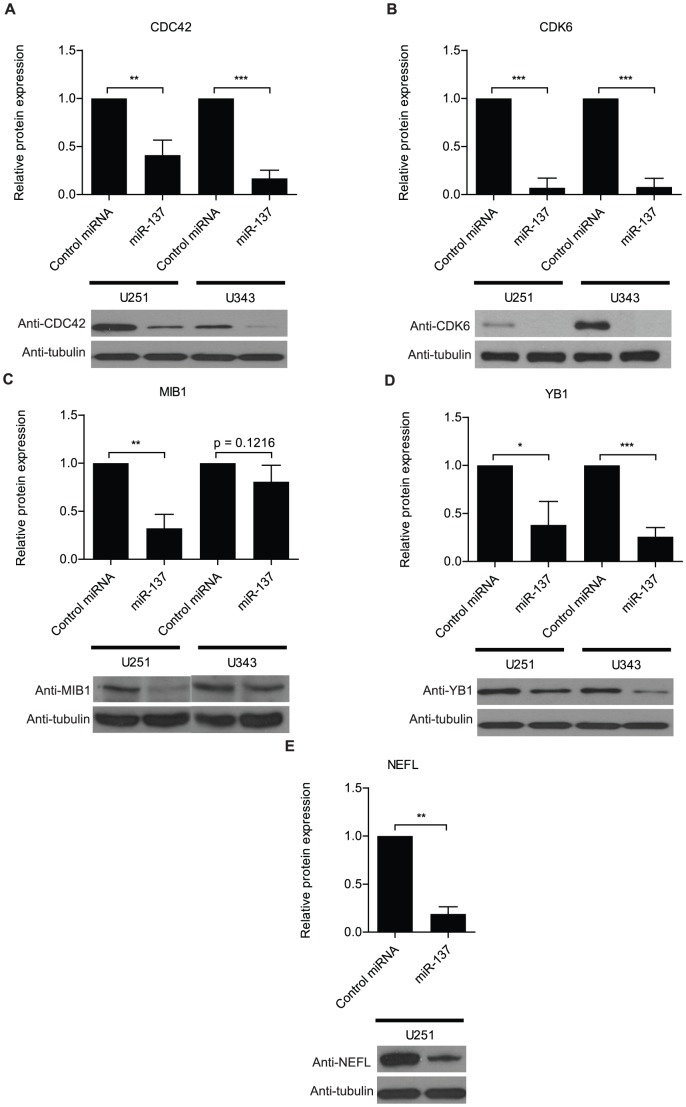
Validation of RNASeq and RIPSeq results by western analysis. U251 and U343 cells were transfected with either miR-137 or control miRNA mimic. Western analyses show the impact on protein levels of a set of genes affected by miR-137 transfection according to RNASeq or RIPSeq analyses. Neurofilament was not detected in U343 cells. α-tubulin was included as an endogenous loading control. Data was analyzed with Student's *t*-test and is presented as the mean ± standard deviation. * indicates p≤0.05, ** indicates p≤0.01, and *** indicates p≤0.001.

To determine if the majority of the identified sets contain miR-137 predicted target genes, we used TargetScan, Pictar and MiRanda to evaluate the number of genes containing miR-137 predicted sites in each set. Out of 647 evaluated genes, 312 (48%) were predicted by at least one tool – **Figure 2B and D**, **Table S1**. TargetScan and MiRanda present differences in predictions while Pictar essentially recognizes a subset of TargetScan predictions - **Figure 2D**, **Table S1**. To determine the significance of our results, we performed a hypergeometric test using the results of each individual prediction tool. These tests produced highly significant results in all cases - **Figure 2C**. Overall, the results suggest that the RIPSeq strategy functions slightly better than straight RNASeq to identify miRNA targets. Identical experiments were performed in U343 cells with similar outcome (**Figure 2** and **Table S1**). Combined, the two analyses identified a total of 595 affected genes containing miR-137 predicted sites, of which 152 genes were identified in both cell lines. This combined set was used in all subsequent bioinformatics analyses described below. To validate our data, a subset of identified targets was analyzed by western blots, qRT-PCR, and luciferase assays – **Figure 3**, **S3**, and **S4**.

Using the computational tools PITA [37] as well as RNAfold [38]), we analyzed structural tendencies of miR-137 binding sites on identified target mRNAs as well as the average participation of nucleotides in base pairing. Most positions on the miR-137 binding region correspond to a less-than-average amount of structure –**Figure S5A**; the only positions that have a significantly higher-than-average tendency of participating in intra-mRNA base pairing correspond to miR-137 positions 6, 7 and 17. These tendencies reflect the fact that microRNA seed region binding is crucial for correct microRNA target recognition. **Figure S5B** shows the fraction of times, that a nucleotide of miR-137 interacts with its target mRNA via base pairing (according to computational predictions based on the RNAduplex program [38]). Nucleotide positions 3–8, corresponding to the miR-137 seed region, are predicted to be more frequently involved in microRNA-mRNA base pairing compared to other regions of the microRNA.

### Understanding the potential impact of miR-137 on gliomagenesis

A series of analyses was conducted to explore the gene set affected by miR-137. First, we performed a literature search with Pubmed to determine, among the identified targets, which genes with oncogenic functions have been previously associated with gliomagenesis. c-KIT, Msi1, AKT2, AEG-1, TGF-ß2, CD24, CDC42, CDK6 and YBX1 were the ones considered most relevant based on function and citations – **Table 1**. This result by itself reveals that miR-137 can have dramatic consequences to the cell, with a robust repression of oncogenic activity. As AKT2 was identified as a miR-137 target, we further investigated its impact on the PI3K/Akt pathway. AKT2 regulation by miR-137 was corroborated by western blot and a reduction in pAkt levels upon miR-137 transfection was observed as well. These results suggest that miR-137 functions as an inhibitor of the PI3K/Akt pathway –**Figure S6**.

**Table 1:** Most relevant oncogenes affected by miR-137 transfection with previously described function in gliomagenesis.

Gene	Function and Association to Glioblastoma	References
AKT2	AKT2 is a putative oncogene that is part of a subfamily of serine/threonine kinases containing SH2-like (Src homology 2-like) domains. AKT2 protein expression increases significantly in high grade gliomas in comparison to low grade gliomas. Reduction of Akt2 in glioma cells inhibited migration, invasion, proliferation and increased cell apoptosis mediated by NFkappaB and BCL2 and chemosensitivity to the anticancer drug VM-26	[78]–[81]
CD24	CD24 is a glycosyl phosphatidylinositol-anchored protein. Glioma tissues with higher grade and lower KPS have significantly higher CD24 expression than those with lower grade and higher KPS, respectively. CD24 stimulates migration of gliomas in vivo and it is possibly implicated in diffuse brain invasion of human gliomas.	[82], [83]
CDC42	CDC42 is a small GTPase of the Rho-subfamily, which controls signaling pathways implicated in cell morphology, migration, endocytosis and cell cycle progression. Depletion of CDC42 abolishes TWEAK-induced Rac1 activation and abrogates glioma cell migration. Slit2 inhibits glioma cell invasion in the brain by suppression of CDC42 activity.	[84], [85]
CDK6	CDK6 is a member of the cyclin-dependent protein kinase (CDK) family. It is part of the protein kinase complex implicated in cell cycle G1 phase progression and G1/S transition. High CDK6 expression and a correlation with prognosis have been observed in gliomas. Amplification of CDK6 was reported in glioma samples. CDK6 knockdown inhibited proliferation and survival of glioblastoma cells and caused significant increase in the apoptosis when cells were treated with temozolomide (TMZ).	[45], [86], [87]
c-kit	c-Kit is a class III receptor tyrosine kinase (RTK) and a proto-oncogene. c-Kit has multiple functions during development, while needed for stem cell maintenance in adult tissue. c-Kit is implicated in many types of cancer providing stem cell characteristics to tumor cells. KIT gene amplification is a common genetic mechanism underlying KIT expression in subset of malignant gliomas.	[88], [89]
MSI1	Msi1 is an RNA binding protein implicated in translation regulation. Msi1 functions as a stem cell marker in a variety of tissues. Its up-regulation was observed in gliomas, medullobastomas, colon cancer, breast cancer, etc. Msi1 high expression was correlated with poor prognosis in the cases of glioma and medulloblastoma. Msi1 regulates a network of targets implicated in cell cycle control, proliferation, apoptosis and cell differentiation.	[9], [90]–[95]
MTDH (AEG-1)	AEG-1 acts as an oncogene that is overexpressed in several types of human cancers, including more than 90% of brain tumors. Its expression is induced by c-Myc oncogene. AEG-1 also interacts with SND1 and involved in RNA-induced silencing complex (RISC) and plays very important role in RISC and miRNA functions. AEG-1 contributes to glioma-induced neurodegeneration through regulation of EAAT2 expression. AEG-1 has the capacity to promote anchorage-independent growth and cooperates with Ha-ras in malignant transformation. Matrix metalloproteases (MMP-2 and MMP-9) are involved in AEG-1-mediated invasion of glioma cells.	[96]–[98]
TGF-β2	Transforming growth factor-beta 2 (TGF-β), is a key factor in the progression of malignant gliomas. TGF-β2 was originally described as “glioblastoma-derived T-cell suppressor factor”, due to its association with the immuno-suppressed status of patients with glioblastoma. High TGF-β2 levels in tumors and in the plasma of patients have been associated with advanced disease stage and poor prognosis. The antisense oligonucleotide trabedersen (AP 12009) that specifically blocks TGF-β2 mRNA has been explored as a therapeutic target.	[99]–[101]
YBX1	YBX1 is a DNA- and RNA-binding protein that interacts with a great variety of proteins. It is involved in processes like DNA replication and repair, transcription, splicing, and mRNA translation. YBX1 is upregulated in many tumors including glioblastoma. YBX1 is highly expressed in the subventricular zone (SVZ) of mouse fetal brain tissues but not in terminally differentiated primary astrocytes. YB-1, Sox-2, Musashi1, Bmi-1, and Nestin were found to be coordinately expressed in SF188 cells and 9/9 GBM patient-derived primary brain tumor-initiating cells (BTIC). Knockdown of YBX1 attenuated the expression of these NSC markers, reduced neurosphere growth, and triggered differentiation via coordinate loss of GSK3-β. YBX1 inhibition also reduced tumor cell invasion and growth in monolayer as well as in soft agar and enhanced temozolomide sensitivity.	[102]–[104]

**Figure 4:**
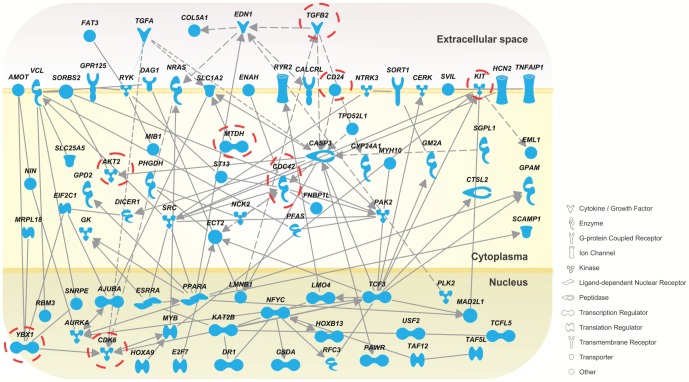
Network analysis. Genes negatively affected by miR-137 mimics transfection as determined by RIPSeq or RNASeq analyses which contain a miR-137 predicted site were connected using Ingenuity Pathway Analysis tool. Solid lines indicate direct interactions; dashed lines indicate indirect interactions. Genes from Table 1 are highlighted (open circle).

A gene ontology analysis identified cell cycle as the main process regulated by miR-137 - **Table S2**. Network analysis was performed with Ingenuity to determine the interactivity among identified miR-137 targets. Proteins with the highest number of connection include TCF3, YBX1, CASP3, CDC42, EDN1 and NFYC – **Figure 4**. Present in this network are several oncogenes listed in **Table 1** (c-Kit, AKT2, TGF-ß2, CD24, CDC42, CDK6 and YBX1).

**Figure 5:**
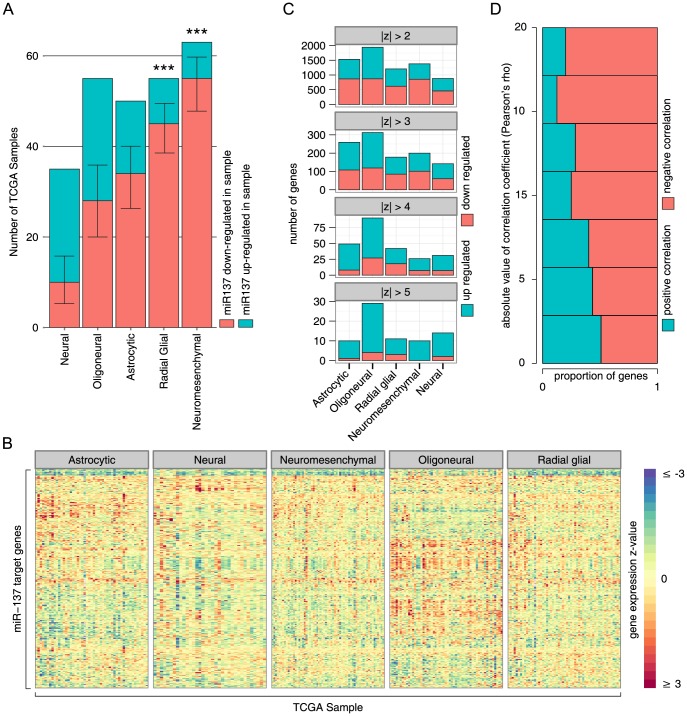
Expression analysis of miR-137 and its targets in the TCGA dataset. **A**) Shown is the total number and proportion of TCGA samples analyzed with up- or down-regulation of miR137 (see methods for details). Error-bars are 95% confidence interval, and *** indicates p≤0.001, binomial test (two tailed, null hypothesis is a true proportion of 0.5). **B**) The z-score of gene expression in 261 TCGA samples for the 595 miR137 target genes relative to normal tissue, stratified by GBM type as reported by Kim et al. [1]. **C**) The number and proportion of up/down regulated genes amongst the 595 identified miR137 target genes in 261 TCGA GBM samples. Four different thresholds for significant change are shown (absolute value of z-score relative to normal tissue greater than 2, 3, 4 or 5). Samples are stratified by GBM type (x-axis), as defined in Kim et al. [1]; if a gene is up or down regulated in multiple samples, it is counted once for each sample showing a significant change. **D**) Proportion of genes with positive or negative correlation with miR137 (x-axis) for increasing correlation coefficient threshold cut-offs (y-axis); higher correlations tend to be predominantly negative.

We finally evaluated the expression profile of miR-137 and its targets using the datasets generated by Kim et al. [1] – **Figure 5**. They defined five GBM subgroups (Astrocytic, Neural, Neuromesenchymal, Oligoneural and Radial Glial). miR-137 shows an overall tendency of down-regulation across the 261 GBM samples present in the TCGA data set when compared to normal tissue. Down-regulation is particularly prevalent in the Radial Glial and Neuromesenchymal subgroups - **Figure 5A**. As expected, the 595 identified miR-137 targets show an opposite pattern in GBM samples with a tendency of up-regulation in relation to normal brain tissue – **Figure 5B–C**. Finally, we examined the expression correlation between miR-137 and identified targets. Although not strong, we observed a trend of negative-correlation (**Figure 5D**), as predictable based on miRNA-mRNA target relationship. Protein expression values would be more appropriate to perform this correlation study since it captures the full extent of miRNA regulation. Unfortunately, due to technical difficulties and sample limitation, genome-wide protein studies have not been performed for these samples.

### miR-137 potentially shares a large number of targets with miR-124, -128 and -7

**Figure 6:**
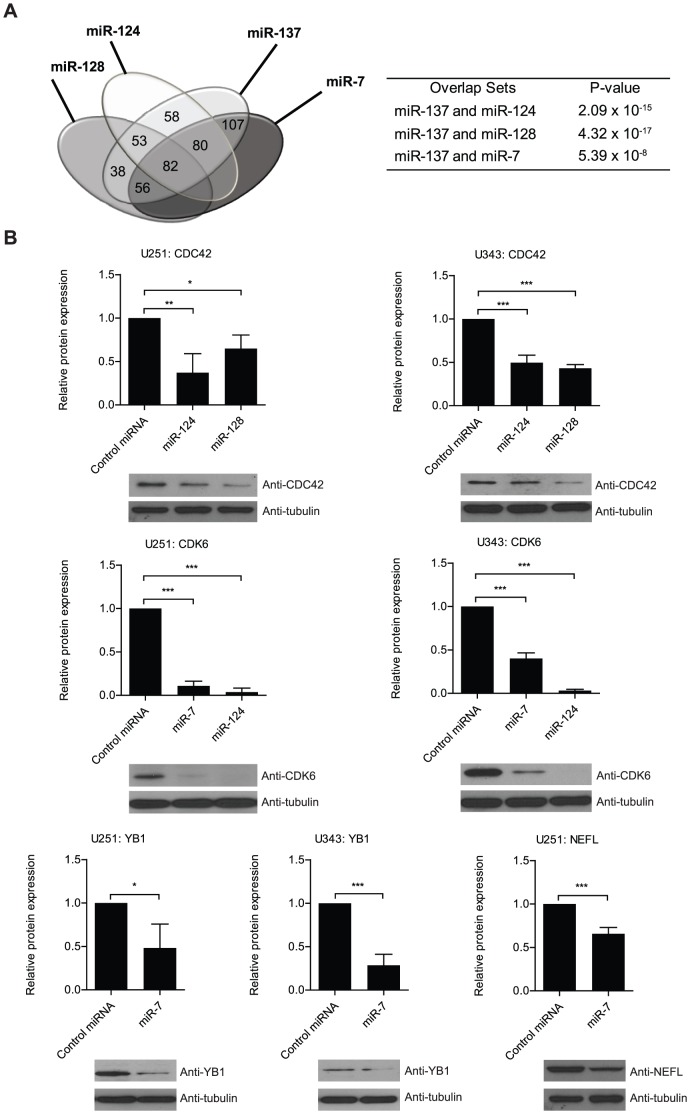
miR-137 potentially shares targets with miRNAs implicated in neurogenesis and gliomagenesis. **A**) Venn diagram displays the potential overlap between miR-137 identified targets and predicted miR-7, -124 and -128 targets obtained from TargetScan, miRanda, and PicTar. Table indicates that overlaps are significant according to Hypergeometric test. **B**) U251 glioblastoma cells were transfected with either miR-7, miR-124, and miR-128 (as listed) or control mimics. Western analyses show the impact on protein levels of a set of genes affected by miR-7, miR-124, or miR-128 transfection that were identified by the bioinformatics analyses. Data was analyzed with Student's *t*-test and is presented as the mean ± standard deviation. * indicates p≤0.05, ** indicates p≤0.01, and *** indicates p≤0.001. Neurofilament was not detected in U343 cells.

Several miRNAs have been shown to be aberrantly expressed in glioblastoma. However, only a small subset of down-regulated miRNAs has been listed by different studies. miR-7, -124 and -128 have been shown to behave similarly to miR-137; meaning that their up-regulation is apparently required for neuronal differentiation while its down-regulation is associated with gliomagenesis [4]–[6]. Thus, we suggest that miR-137 may act in a coordinated manner with these three miRNAs to boost the regulatory effect on critical targets during neurogenesis. On the other hand, their simultaneous down-regulation could produce a robust change in the expression of important oncogenic proteins. Predicted targets of miR-7, -124 and -128 were compiled from TargetScan, Miranda and Pictar and compared to our miR-137 list. Results showed strong overlap; out of the 595 genes containing miR-137 sites obtained with our analyses, 82 (∼14%) are potentially targeted by all 4 miRNAs while 189 targets (∼32%) are in theory shared by miR-137 and other two miRNAs – **Figure 6A** and **Table S3**. All the results are statistically significant. Moreover, we determined that among the critical glioblastoma players listed in **Table 1**, several of them including c-KIT, TGFβ2, CDK6, AKT2, LRRC4, YBX1, CD24 and MTDH are potentially targeted by at least two of the mentioned miRNAs. A set of predicted targets was validated by western blot - **Figure 6B**.

## Discussion

Several approaches are available to identify miRNA targets, starting with multiple target prediction methods and continuing with a variety of biological procedures that include reporter based screenings, shotgun proteomics, transcriptomic analyses and Ago2 based immunoprecipitation methods [39]–[44]. Although *in silico* predictions are becoming more accurate, they will never substitute biological methods. No single approach is comprehensive enough; the advantages of employing dual approaches can be illustrated by the miR-122 target analysis that we conducted using luciferase-based screening in combination with APEX shotgun proteomics [44]. In the current study, we also used a dual strategy to evaluate the impact of miR-137 in glioblastoma cells. The novelty was the usage of PABP as a reporter of miRNA activity. This concept is in line with recent results from Mathias Hentze's group that show PABP as a central player in miRNA function. In their model, PABP has a dual role: PABP and the poly A tail enhance binding of miRISC to the target mRNA during early stages of silencing, then PABP is later displaced by miRISC, allowing recruitment of deadenylase complexes and subsequent deadenylation of target mRNA [24]. Therefore, a decrease in association of PABP with mRNAs upon miR-137 action could be a direct measurement of its activity.

Many published studies attempt to justify the involvement of tumor suppressor miRNAs or oncomiRs in tumorigenesis based on their action on a small set of genes or sometimes a single target. In most cases if not all, the conclusions are an oversimplification of a quite complex scenario. For instance, we have previously shown that miR-122 downregulation potentially affects the development of hepatocellular carcinoma (HCC) by regulating multiple genes associated with processes like apoptosis, cell cycle, cell death, cell differentiation, cell growth, cell proliferation and mitosis, including Vimentin, CREB1, SEPT2, PKM2 and CREB [44]. In this current work, we showed that miR-137 transfection in glioblastoma cells trigger the down-regulation of a collection of oncogenic genes. This observation is in alignment with results indicating that transfection of miR-137 mimics in glioma cells leads to a decrease in proliferation, cell cycle arrest in G_0_/G1 phase, affects xenograft and colony growth and invasion [6], [11], [45]. The most relevant oncogenes affected by miR-137 transfection are discussed in **Table 1**. Other cancer relevant genes that are worth mentioning include TCF3, a member of the E protein (class I) family of helix-loop-helix transcription factors which functions as a key regulator of stem cell pluripotency and differentiation via the Wnt pathway [46]; DCLK1 is a serine/threonine-protein kinase that binds microtubules and regulates its polymerization [47] and is implicated in neurogenesis, neuronal migration, transport, and apoptosis [48]–[51]; MIB1 is an E3 ubiquitin ligase that regulates endocytosis of Notch ligands [52] with potential roles in the formation of late neural progenitors mainly designated for gliogenesis [53] and tumor progression [54]–[58]. RYK is a member of the family of growth factor receptor protein tyrosine kinases required for Wnt pathway activation in partnership with MIB1 [59]. SEMA4D belongs to class IV semaphorin, it is highly expressed in embryonic nervous system and involved in a variety of processes that include tumor angiogenesis, invasiveness and regulation of tumor-associated macrophages [60]; NRAS is an oncogene encoding a membrane protein (GTPase) that shuttles between the Golgi apparatus and the plasma membrane [61]. SRC and the Src family of proteins have been extensively characterized to have important roles in the proliferative and invasive abilities in many cancers including glioblastoma [62]. Src seems to be activated when epidermal growth factor signaling is elicited, resulting in enhanced tumor progression and invasion [63]. More recently, studies of Src have focused on the development of targeted therapy for cancer treatment. Dasatinib has been tried as a possible therapy against Src in glioblastoma [64]. The use of dasatinib in experimental murine xenograft and orthotopic models results in inhibition of bevacizumab-induced glioma cell invasion [65]. AQP4 (aquaporin-4) belongs to the aquaporin family of water-channel proteins. Aquaporin-4 have normal roles in brain tissue, regulating the brain water homeostasis, acting on the blood-brain barrier and brain-cerebrospinal fluid [66]. In gliomas, aquaporin-4 is upregulated [67], and this upregulation is thought to result in increased migration/invasion as well as contributing to tissue edema [68], [69].

Transport is a biological process enriched among miR-137 affected genes in glioblastoma cells; in this category, we identified several members of the solute carrier family (SLC) (SLC16A9, SLC1A2, SLC1A5, SLC25A5 SLC35A4, SLC43A2, SLC45A3, SLC6A8, SLC4A7, SLC7A2) which codes for membrane transport proteins. They are responsible for the cellular uptake of a broad range of endogenous compounds and associated with drug-resistance in cancer [70]. SLCs have been implicated in neurological disorders, including schizophrenia. Interestingly, single nucleotide polymorphism in the miR-137 gene is highly associated with schizophrenia [71].

The association between Musashi1 (Msi1) and miR-137 is particularly interesting. We identified Msi1 as a target of miR-137 in a previous work [9] and in this current study. The regulation of Msi1 by miR-137 is highly conserved as the target site is not only present in multiple vertebrates but also in the *Drosophila* orthologues Musashi and RBP6. Msi1 and miR-137 have apparently antagonistic roles and opposite expression patterns in neurogenesis and brain tumor formation. Msi1 is a stem cell related RNA binding protein implicated in stem cell self-renewal and development of both medulloblastoma and glioblastoma. A decrease in Msi1 expression is required for neuronal differentiation while its high expression is frequently associated with tumors (reviewed in [72]). Recent CLIP analysis done in our lab to identify Msi1 targets in U251 cells demonstrated that the association between these two regulators goes beyond the direct regulation of Msi1 by miR-137. Msi1 and miR-137 share 83 targets (p<1.87×10^−10^; Fisher's exact test) and might regulate their expression in opposite directions. Moreover, when assembled together, Msi1 and miR-137 identified targets produced a complex gene network. Therefore, we suggest these two regulators function as opposite nodes of a post-transcriptional network implicated in gliomagenesis and neurogenesis (Vo DT et al., *submitted*).

It is well established that a given mRNA can be targeted by multiple miRNAs [73]. These associations occur genome-wide and can lead to the formation of complex biological networks. The prediction of miRNA-miRNA synergistic networks based on common targets of corresponding miRNA pairs, enriched in the same gene ontology category and close proximity in protein interaction network showed that an extensive cross-talk takes place among different miRNA species. Interestingly, miRNAs associated with the same disease are close to each other in projected networks [74]. Our data is in agreement with this idea and suggests that miR-137 potentially shares the same group of mRNA targets with miR-7, -124 and -128 to drive cells towards neurogenesis and while absent, these miRNAs can produce a strong oncogenic response that could contribute to gliomagenesis.

Much of the great interest in miRNAs is related to their potential use in cancer therapy. The broad impact on gliomagenesis revealed by our study suggests that miR-137 mimics could become a viable alternative to treat brain tumor patients. As stated earlier, miR-137 expression is lost, putatively during gliomagenesis suggesting that it acts as a tumor suppressor, acting by repressing the expression of oncogenes such as those identified in this paper. Replenishment of miR-137 provides an opportunity to broadly repress these oncogenes and cancer-relevant genes. Use of mimics and antagomiRs as part of cancer therapeutic treatment has been discussed intensively and several investigators are already exploring approaches for *in vivo* delivery and running assays in murine systems [75]–[77]. However, successful strategies tested in other tumors might fail in glioblastoma due to the brain blood barrier, necessitating the exploration of alternative delivery methods such as nanoparticles and other carriers.

## Supporting Information

Methods S1 (DOC)

Figure S1 **A RIPSeq-based approach to identify miRNA target genes.** **A**) The idea behind this approach is that PABP has major roles in miRNA function, first it helps RISC to identify its target site and second, PABP is later displaced by miRISC, allowing recruitment of deadenylase complexes and subsequent deadenylation of target mRNA [24]. **B**) Summary of experimental approach to identify miRNA targets using PABP as a reporter. Transfected miR-137 mimics will disrupt the interaction between PABP and its target mRNAs, decreasing the levels of immuno-precipitated mRNAs; the read-out would be a reduction in the number of sequence reads for the affected mRNAs.
(EPS)

Figure S2 **Target identification of miR-137 by RNASeq and RIPSeq produced markedly different target sets.** **A**) The log fold-change for those transcripts identified in the total-RNA sample (left two boxes) when examined in the IP samples (blue box) is on average below the established threshold (dashed line) with many showing little change, or a change in the opposite direction. The same comparison is shown in the right two boxes for those targets identified in the IP sample. **B**) Histograms showing the difference in average fold-change between IP and Total-RNA samples for those targets identified only in the total-RNA (left) and the IP (right). In both cases, close to half of the targets identified show more than 1× greater decrease in fold-change for the method where the target was identified over that where it was not – e.g. 1× difference in IP (i.e. no change) vs. 2× in Total RNA would be a 1× difference.
(EPS)

Figure S3 **Validation of targets identified by RNASeq and RIPSeq by quantitative reverse transcription and polymerase chain reaction (qRT-PCR).** U251 and U343 cells were transfected with either miR-137 or control mimics. qRT-PCR analyses show the impact on mRNA levels of a set of genes affected by miR-137 transfection according to RNASeq or RIPSeq analyses. Data was analyzed with Student's *t*-test and is presented as the mean ± standard deviation. * indicates p≤0.05 and ** indicates p≤0.01.
(EPS)

Figure S4 **Validation of RNASeq and RIPSeq results by luciferase.** Luciferase assay with 3′ UTR constructs of miR-137 identified targets co-transfected with miR-137 or control mimics. Both original 3′ UTRs and 3′UTRs containing deletions of predicted seed sequences (Δ) were analyzed. Experiment was performed in triplicate. Data was analyzed using the Student's t-test and shown as mean ± standard deviation. * denotes P≤0.05 and ** denotes P≤0.01.
(EPS)

Figure S5 **Characteristics of the miR-137 binding motif.** **A**) Shown is the average probability of intra-mRNA base pairing for a set of mir-137 binding sites as a function of nucleotide position on the mRNA. Position -1 corresponds to the 5′ end of a predicted microRNA binding site on an mRNA and position 1 of the microRNA guide strand. Note that the precise extent of the microRNA binding site might vary between binding sites. **B**) The fraction of times that a particular nucleotide on mir-137 is participating in miRNA-mRNA binding is estimated for a set of binding sites using the RNAduplex program. Position 1 corresponds to the 5′ end of the guide strand (and the 3′ end of the microRNA binding site on the mRNA).
(EPS)

Figure S6 **miR-137 and Akt pathway activation.** Western analysis of U251 cells transfected with control and miR-137 mimics shows that miR-137 inhibits Akt pathway activation as reflected by a reduction in Akt and pAkt. ß-actin was included as an endogenous loading control.
(EPS)

Table S1 **Complete list of genes affected by miR-137 mimics transfection.** Table lists genes identified in both approaches (RIPSeq and RNASeq), the presence of a miR-137 sites according to TargetScan, MiRanda and Pictar and if the gene was previously characterized as a target in other study.
(XLSX)

Table S2 **Gene ontology of identified miR-137 targets.**
(XLSX)

Table S3 **Overlap between miR137 identified targets and predicted targets for miR-124, -128 and -7 according to TargetScan, MiRanda and Pictar.**
(XLSX)
