# Supplementary material for: Genomic Analyses Reveal Broad Impact of miR-137 on Genes Associated with Malignant Transformation and Neuronal Differentiation in Glioblastoma Cells
Source: PLoS One. 2014 Jan 22;9(1):e85591. doi: 10.1371/journal.pone.0085591 (PMC3899048; doi:10.1371/journal.pone.0085591)
Supplement: Methods S1 — (DOC) [file pone.0085591.s001.doc]

**Supplementary Methods**

**Building the miR-137 binding motif**

Binding site characterization was based on a set of binding sites predicted by the PITA program applied to a set of 3’UTR regions of 1090 distinct human mRNA sequences that were identified experimentally as mir-137 targets [1]. The predicted free energy of binding was required to be (according to PITA predictions) less than or equal to -5kcal/mol (318 predicted sites on 267 distinct mRNAs) and -10kcal/mol (23 predicted sites on 23 distinct mRNAs) [2]. These data sets are referred to as the -5kcal/mol binding site data set and the -10kcal/mol binding site data set respectively. The average amount of intra-mRNA base pairing was estimated by running the RNAfold program using its options for exporting a probability matrix [2]. For each nucleotide position of a predicted binding site, the probability, that a certain nucleotide of the mRNA is participating in base pairing, is averaged over the set of all considered predicted binding sites [2]. The fraction of times that a particular nucleotide on the mir-137 is participating in miRNA-mRNA binding is estimated using the RNAduplex program applied to regions that were predicted to be microRNA binding sites according to the PITA program (sites with predicted free energy of binding less than -10kcal/mol) [1,2].

**References**

1. 1. Kertesz M, Iovino N, Unnerstall U, Gaul U, Segal E (2007) The role of site accessibility in microRNA target recognition. Nat Genet 39: 1278-1284.
2. 2. Lorenz R, Bernhart SH, Honer Zu Siederdissen C, Tafer H, Flamm C, et al. (2011) ViennaRNA Package 2.0. Algorithms Mol Biol 6: 26.
